# Association between early childhood oral health impact scale (ECOHIS) scores and pediatric dental surgery wait times

**DOI:** 10.1186/s12903-020-01263-8

**Published:** 2020-10-17

**Authors:** Victor H. K. Lee, Cameron G. Grant, Betty-Anne Mittermuller, Sarbjeet Singh, Brenda Weiss, Jeanette M. Edwards, Robert J. Schroth

**Affiliations:** 1 Department of Preventive Dental Science, Dr. Gerald Niznick College of Dentistry, Rady Faculty of Health Sciences, University of Manitoba, 507 – 715 McDermot Avenue, Winnipeg, Manitoba R3E 3P4 Canada; 2 Children’s Hospital Research Institute of Manitoba, Winnipeg, Manitoba Canada; 3 Shared Health, Winnipeg, Manitoba Canada; 4 Department of Pediatrics and Child Health, Max Rady College of Medicine, Rady Faculty of Health Sciences, University of Manitoba, Winnipeg, Manitoba Canada; 5 Section of Pediatric Dentistry, Winnipeg Regional Health Authority, Winnipeg, Manitoba Canada

**Keywords:** Early childhood caries, Preschool child, ECOHIS, Oral health-related quality of life, wait times, COVID-19

## Abstract

**Background:**

Severe Early Childhood Caries (S-ECC) is an aggressive form of tooth decay that often requires pediatric dental rehabilitative surgery. The Early Childhood Oral Health Impact Scale (ECOHIS) measures oral health-related quality of life (OHRQL). The purpose of this study was to determine whether there is an association between ECOHIS scores and surgery wait times for children undergoing dental treatment for S-ECC under general anesthesia (GA).

**Methods:**

The hypothesis was that there is no present association between wait times and ECOHIS score. Children under 72 months of age with S-ECC were recruited on the day of their slated dental surgery under GA. Parents/caregivers completed a questionnaire that included the ECOHIS. Data were merged with other ECOHIS scores from a previous study. Wait times were acquired from the Patient Access Registry Tool (PART) database. Data analysis included descriptive statistics and bivariate analyses. A *p*-value of ≤0.05 was considered statistically significant; 95% confidence intervals (CIs) were reported for each correlation coefficient. This study was approved by the University of Manitoba’s Health Research Ethics Board.

**Results:**

Overall, 200 children participated, the majority of whom were Indigenous (63%) and resided in Winnipeg (52.5%). The mean age was 47.6 ± 13.8 months and 50.5% were female. Analyses showed ECOHIS scores were not significantly correlated with children’s wait times. Observed correlations between ECOHIS and children’s wait times were low and not statistically significant, ranging from ρ = 0.11 for wait times and child impact section (CIS) scores (95% CI: − 0.04, 0.26; *p* = 0.14), ρ = − 0.08 for family impact section (FIS) scores (95% CI: − 0.23, 0.07; *p* = 0.28), and ρ = 0.04 for total ECOHIS scores (95% CI: − 0.11, 0.19; *p =* 0.56).

**Conclusion:**

No significant associations were observed between ECOHIS scores and wait times. In fact, those with worse OHRQL appeared to wait longer for surgery. ECOHIS scores could, however, still be used to help prioritize children for dental surgery to ensure that they receive timely access to dental care under GA. This is essential given the challenges posed by COVID-19 on timely access to surgical care.

## Background

Early Childhood Caries (ECC) is a chronic disease of childhood that is defined as dental decay affecting the primary dentition in children under 72 months of age [1]. A considerable proportion of children are disproportionately affected by a more aggressive form of ECC, called Severe ECC (S-ECC) [2–4]. The prevalence of ECC among young children is of concern as severe forms can negatively affect health and well-being [2, 5, 6]. ECC among priority groups (i.e., low socioeconomic status, rural and remote dwelling, Indigenous peoples, refugees and newcomers) is becoming a public health concern [7]. In some northern and remote communities in Canada, up to 90% of children are affected [8, 9]. For many, rehabilitative dental surgery under general anesthesia (GA) is the only option due to the severity of their condition [10]. In the province of Manitoba, over 3000 children under 6 years of age receive dental surgery under GA in hospitals each year, and approximately 1600 of these cases are performed in Winnipeg Regional Health Authority (WRHA) facilities or WRHA-funded facilities [11].

Long wait times for pediatric dental rehabilitative surgery is a national problem [11–13]. At the present time, the wait list for dental surgery at the Misericordia Health Centre in Winnipeg, Canada in 2018–2019 and 2019–2020 has ranged between 8.9 and 17.0 weeks (average 11.9 weeks). The Pediatric Canadian Access Targets for Surgery (P-CATS), a diagnosis-based access targets priority classification scheme, states that children requiring rehabilitative dental surgery should be seen within 13 weeks from the decision made to proceed with surgery [14]. Unfortunately, there is no formal prioritization system for triaging individual cases that is universally used or implemented for use by pediatric dentists in Manitoba and the rest of Canada. The disruption to dental services because of COVID-19 further highlights the need for prioritization of cases (based on objective criteria) on growing surgical wait lists.

**Fig. 1:**
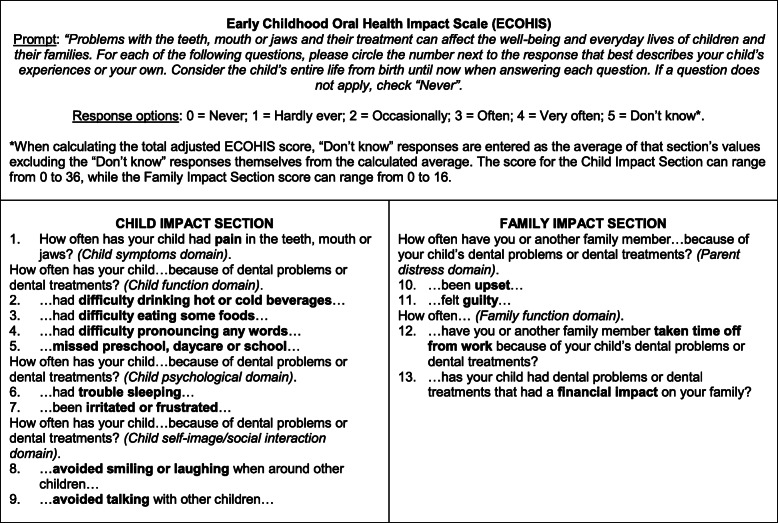
The Early Childhood Oral Health Impact Scale (ECOHIS)

S-ECC is recognized to affect multiple aspects of childhood health [2]. Oral health-related quality of life (OHRQL) is also affected as these children are known to exhibit pain, altered eating habits and sleeping patterns, and behaviour changes [15]. Children’s OHRQL could therefore indicate the severity of S-ECC and the level of S-ECC-related suffering. The Early Childhood Oral Health Impact Scale (ECOHIS) (Fig. 1) is a validated tool for use with parents or caregivers to assess young children’s OHRQL (under 72 months of age) [16]. ECOHIS could significantly help prioritize children waiting for surgery based on the impact of caries on childhood OHRQL, so that children with a higher need of surgery are able to access care and minimize the wait time for surgery or access other forms of dental care that may be more appropriate at their age.

Our current hypothesis is that there presently is no association between children’s wait times for pediatric dental rehabilitative surgery and ECOHIS scores. The primary objective of this exploratory study was to determine whether there currently is an association between children’s ECOHIS scores and pediatric dental rehabilitative surgery wait times.

## Methods

Eligibility for this study was restricted to children with S-ECC undergoing pediatric dental rehabilitative surgery using GA and their parent(s) or caregiver(s). In keeping with the recognized definition of S-ECC [1], only children under the age of 72 months were recruited into this study. Data on ECOHIS scores and wait times were obtained in two ways: 1) by recruiting children and their parents on the day of the child’s scheduled dental surgery at the Misericordia Health Centre, and 2) by accessing ECOHIS scores from children already enrolled into an existing study on nutritional status and well-being changes in children after dental surgery [15]. Both studies were approved by the Health Research Ethics Board at the University of Manitoba (HS20846/H2017:187 and HS18663/H2015:237).

A convenience sample of 50 children undergoing pediatric dental rehabilitative surgery at the Misericordia Health Centre to treat S-ECC were recruited into this study. There was no difference in the recruitment and data collection processes from the larger study (described in the previous paragraph). Parents and caregivers provided written informed consent and were asked to complete a short questionnaire that included the ECOHIS questionnaire to help assess the OHRQL. ECOHIS is a questionnaire which has construct validity, internal consistency, and reliability for measuring OHRQL in preschool-aged children [16]. The ECOHIS tool includes 13 questions grouped into two parts: the Child Impact Section (CIS) and Family Impact Section (FIS). The CIS has nine questions categorized into four domains: child’s symptoms (one question), child functions (four questions), child psychology (two questions), and child’s self image/social interaction (two questions). The FIS has four questions covering two domains: parental distress (two questions) and family function (two questions) [16].

Following ECOHIS protocol, each question was scored on a scale from 0 to 4 (0 = never, 1 = hardly ever, 2 = occasionally, 3 = often, 4 = very often), and “don’t know” responses were treated as missing. A total score for each child was calculated by summing up response codes, with a maximum total score of 52 [16]. Higher scores indicate a greater negative impact on OHRQL. For surveys with up to two “missing”/“don’t know” responses in the CIS, or one “missing”/“don’t know” in the FIS, a score for the missing items was inputted as an average of the remaining items for that section. If the CIS had more than two “missing responses”, or the FIS had more than one “missing” response, that section was excluded from scoring.

The questionnaire also collected demographic data for the child and family (e.g., age, sex, place of residence, presence and type of dental insurance, etc.). Children’s wait for surgery, defined as the wait time (in weeks) from date of client readiness (date started on wait list) to date of intervention or surgery, were gathered from the Patient Access Registry Tool (PART) database and linked with participant’s ECOHIS scores.

Data collected from the 50 children were merged with data from the 150 parent-child dyads that had been recruited into the existing study. Parents and caregivers completed the ECOHIS at the time of their child’s dental surgery at the Misericordia Health Centre as part of that study’s protocol as well. The need for additional consent to use the ECOHIS data already obtained in this study was waived as the children’s wait times for dental surgery from the PART database were merely added in. The wait times for these 150 children in study (HS18663/H2015:237) were obtained and linked with corresponding ECOHIS scores. Power calculations conducted by the authors showed that, anticipating a correlation of ≥0.20 and applying an alpha of 0.05, a sample of size of 194 children would have been required to achieve 80% power.

Data were entered into an Excel spreadsheet and analyzed using Number Cruncher Statistical Software Version 12 (NCSS, Kaysville, Utah). Descriptive statistics included frequencies and means ± standard deviation (SD). Bivariate analyses included *t*-tests and Spearman’s correlation. Correlation analyses for wait times was performed using the ECOHIS child impact section (CIS) scores, the ECOHIS family impact section (FIS) scores, and total ECOHIS scores as covariates. A *p* ≤ 0.05 was considered significant; 95% confidence intervals (CIs) were reported for each correlation coefficient.

## Results

A total of 200 children participated; with a mean age of 47.6 ± 13.8 months and 50.5% being female. Wait times data were available for 174 children as some had missing wait time information from the PART database (Table 1). Nearly 60% of children underwent surgery within the 12.9 week (3 month) wait time target suggested by the P-CATS. The mean wait time was 14.3 ± 15.0 weeks, ranging from 0.1 weeks to 133.7 weeks. Almost all participants (92%) had some form of dental insurance that covered all or part of their dental expenses. Most participants (76%) were covered by a government program, including the non-insured health benefits (NIHB) program for registered First Nations persons. Registered First Nations persons are those individuals who have legal status under the *Indian Act* of Canada. These individuals have certain rights and benefits that are not available to non-status First Nations, Métis, Inuit, or other Canadians. The majority of participants (63%) were registered First Nations and half (52.5%) resided in Winnipeg, Canada,

**Table 1:** Characteristics of Participants

VARIABLE	TOTAL ***N*** = 200 (% IN BRACKETS)^**a**^
**Sex of child**	
Male	99 (49.5)
Female	101 (50.5)
Child’s mean age, months ± standard deviation	47.6 ± 13.8
**Resides in Winnipeg**	
Yes	104 (52.5)
No	94 (47.5)
Wait time of < 13 weeks	
Yes	104 (59.8)
No	70 (40.2)
Child’s mean wait time, weeks ± standard deviation	14.3 ± 15.0 (Range 0.1–133.7)
**Registered First Nations**	
Yes	126 (63.0)
No	74 (37.0)
**Overall health rating by parent**	
Very good	102 (51.0)
Good	86 (43.0)
Fair	12 (6.0)
**Medical/health problems**	
Yes	31 (15.5)
No	169 (84.5)
**Medications**	
Yes	16 (8.0)
No	184 (92.0)
**Parent/caregiver education**	
<High school	56 (29.5)
High school	76 (40.00)
Some college/university	58 (30.5)
**Receives government/social assistance**	
Yes	107 (53.5)
No	88 (44.0)
Don’t Know	5 (2.5)
**Household income**	
<$28,000	108 (54.0)
>$28,000	59 (29.5)
Don’t Know	33 (16.5)
**Dental insurance**	
Yes	184 (92.0)
No	8 (4.0)
Don’t Know	8 (4.0)
**Type of insurance**	
None	16 (9.0)
Government insurance	152 (76.0)
Private insurance	32 (16.0)

Note: Participants chose not to answer some questions (i.e. not all questions have 200 responses)

^a^Except where other units are given

Due to dental problems or treatments, 40% of children experienced pain in their teeth, mouths of jaws at least occasionally or more frequently (Table 2). Further several children (26.5%) had difficulty eating foods occasionally or more frequently, while 18% had difficulties drinking hot or cold beverages occasionally or more frequently. Overall, 26% of children were reported to either occasionally, often or very often experience irritation or frustration due to dental problems or treatments, and 13% had difficulties pronouncing words (Table 2). Several children (14.5%) occasionally or often had trouble sleeping due to dental problems or treatments, and 2.5% had to miss school occasionally or more frequently because of dental problems or dental treatments. Almost all children never avoided smiling and laughing (95.5%) or talking (96%) when around other children because of dental problems or treatments.

**Table 2:** Early Childhood Oral Health Impact Scale Response Summary (Total *N* = 200 [% in brackets])

ECOHIS Questions	Mean (0–5)	0 = Never	1 = Hardly Ever	2 = Occasionally	3 = Often	4 = Very Often	5 = Don’t Know
**1. PAIN**	1.24 ± 1.20	74 (37.0)	45 (22.5)	51 (25.5)	21 (10.5)	8 (4.0)	1 (0.5)
***Child symptoms domain mean score ± SD*:**				1.2 ± 1.2			
**2. DRINKING**	0.52 ± 0.98	148 (74.0)	16 (8.0)	22 (11.0)	12 (6.0)	2 (1.0)	0 (0.0)
**3. EATING**	0.83 ± 1.20	121 (60.5)	24 (12.0)	32 (16.0)	16 (8.0)	5 (2.5)	2 (1.0)
**4. PRONOUNCING**	0.54 ± 1.20	157 (78.5)	12 (6.0)	12 (6.0)	9 (4.5)	5 (2.5)	5 (2.5)
**5. ABSENCE**	0.43 ± 0.96	144 (72.0)	45 (22.5)	4 (2.0)	1 (0.5)	0 (0.0)	6 (3.0)
***Child function domain mean score ± SD*:**				2.1 ± 2.5 (0–12)			
**6. SLEEPING**	0.40 ± 0.78	153 (76.5)	18 (9.0)	25 (12.5)	4 (2.0)	0 (0.0)	0 (0.0)
**7. FRUSTRATION**	0.77 ± 1.16	127 (63.5)	21 (10.5)	29 (14.5)	17 (8.5)	6 (3.0)	0 (0.0)
***Child physiological domain mean score ± SD*:**				1.2 ± 1.6 (0–6)			
**8. SMILING**	0.08 ± 0.43	191 (95.5)	5 (2.5)	2 (1.0)	1 (0.5)	1 (0.5)	0 (0.0)
**9. TALKING**	0.09 ± 0.46	192 (96.0)	2 (1.0)	4 (2.0)	1 (0.5)	1 (0.5)	0 (0.0)
***Child self-image/social interaction domain mean score ± SD*:**				0.2 ± 0.7 (0–8)			
**Child Impact Section (CIS) Mean Score ± SD:**				4.6 ± 4.8 (0–28)			
**10. UPSET**	0.84 ± 1.19	120 (60.0)	25 (12.5)	32 (16.0)	14 (7.0)	9 (4.5)	0 (0.0)
**11. GUILTY**	1.09 ± 1.33	103 (51.5)	25 (12.5)	37 (18.5)	23 (11.5)	10 (5.0)	2 (1.0)
***Parent distress domain mean score ± SD*:**				1.9 ± 2.1 (0–8)			
**12. TIME OFF WORK**	0.52 ± 0.81	130 (65.0)	44 (22.0)	19 (9.5)	7 (3.5)	0 (0.0)	0 (0.0)
**13. FINANCIAL**	0.15 ± 0.54	183 (91.5)	9 (4.5)	5 (2.5)	2 (1.0)	1 (0.5)	0 (0.0)
***Family function domain mean score ± SD*:**				0.7 ± 1.0 (0–5)			
**Family Impact Section (FIS) Mean Score ± SD:**				2.5 ± 2.6 (0–12)			
**Total Mean Score ± SD:**				7.2 +/- 6.4			

Approximately one-third of parents occasionally or more frequently felt upset (27.5%) and guilty (35%) (Table 2). Furthermore, 13% of parents had to take time off of work occasionally or more frequently, and 4% reported that their child’s dental problem or treatments occasionally or more frequently had a financial impact on the family.

**Fig. 2:**
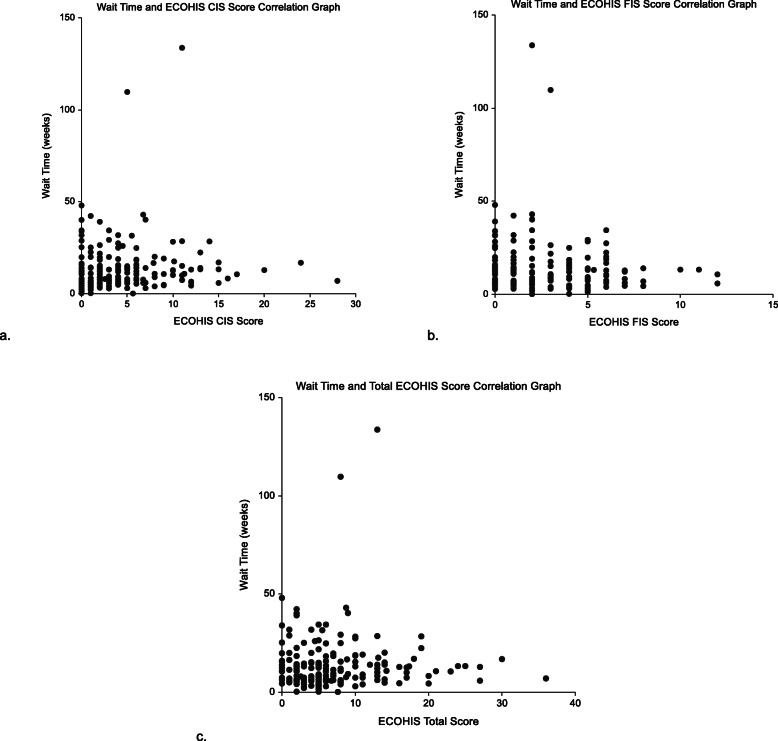
**a** Correlation between ECOHIS CIS scores and pediatric dental surgery wait times (ρ = 0.11; 95% CI: − 0.04, 0.26; *p* = 0.14); **b** Correlation between ECOHIS FIS scores and pediatric dental surgery wait times (ρ = − 0.08; 95% CI: − 0.23, 0.07; *p* = 0.28); **c** Correlation between total ECOHIS scores and pediatric dental surgery wait times (ρ = 0.04; 95% CI: − 0.11, 0.19; *p* = 0.56)

Overall, among the 174 children with complete wait times and ECOHIS data, no significant associations were observed between participants’ ECOHIS scores and dental surgery wait times (Fig. 2). Analyses of ECOHIS CIS scores and wait times revealed a statistically insignificant correlation (ρ = 0.11; 95% CI: − 0.04, 0.26; *p* = 0.14). ECOHIS FIS scores were insignificantly correlated with wait times (ρ = − 0.08; 95% CI: − 0.23, 0.07; *p* = 0.28). Spearman’s correlation results also showed an insignificant correlation between participants’ total ECOHIS scores and wait times (ρ = 0.04; 95% CI: − 0.11, 0.19; *p* = 0.19). In fact, those with worse OHRQL appeared to wait longer for surgery.

A sensitivity analysis conducted by removing two significant outliers (Fig. 2) from analyses did not substantially change results. All values of interest still remained non-significant. Higher CIS scores were once again not significantly correlated with longer wait times (ρ = 0.10; 95% CI: − 0.05, 0.24; *p* = 0.20), lower FIS scores were not significantly correlated with shorter wait times (ρ = 0.10; 95% CI: − 0.24, 0.06; *p* = 0.24), and lower total ECOHIS scores were not significantly correlated with shorter wait times (ρ = 0.03; 95% CI: − 0.12, 0.18; *p* = 0.73).

There was no difference in wait times for surgery, ECOHIS CIS scores, and total ECOHIS scores regardless of place of residence, sex, and of whether or not children underwent surgery within the 12.9 week (3 month) wait time target suggested by the P-CATS (Table 3). The only significant difference in results was observed in ECOHIS FIS scores between those living in rural areas of Manitoba and those living in the city of Winnipeg (3.0 ± 2.6 vs. 2.2 ± 2.6, *p* = 0.04).

**Table 3:** Participant Group Status and Wait Time and ECOHIS Score Summary

PARTICIPANT GROUP	MEAN VALUE ± SD	p
**Wait time (weeks) for surgery:**		
Registered First Nations	15.1 ± 17.7	0.37
Non-First Nations	13.0 ± 8.9	
Rural	14.0 ± 15.3	0.65
Urban (Winnipeg)	14.7 ± 14.5	
Male	14.5 ± 14.2	0.83
Female	14.0 ± 15.8	
**ECOHIS child impact section (CIS) score:**		
Registered First Nations	4.3 ± 4.8	0.24
Non-First Nations	5.1 ± 4.8	
Rural	4.6 ± 4.5	0.95
Urban (Winnipeg)	4.7 ± 5.0	
Male	5.1 ± 4.7	0.18
Female	4.2 ± 4.8	
> 13 week wait time	4.5 ± 4.9	0.51
< 13 week wait time	4.9 ± 4.7	
**ECOHIS family impact section (FIS) score:**		
Registered First Nations	2.4 ± 2.4	0.26
Non-First Nations	2.8 ± 2.9	
Rural	3.0 ± 2.6	**0.037**
Urban (Winnipeg)	2.2 ± 2.6	
Male	2.4 ± 2.8	0.60
Female	2.6 ± 2.4	
> 13 week wait time	2.3 ± 2.6	0.32
< 13 week wait time	2.8 ± 2.7	
**Total ECOHIS score:**		
Registered First Nations	6.7 ± 6.1	0.18
Non-First Nations	7.9 ± 6.7	
Rural	7.6 ± 6.0	0.43
Urban (Winnipeg)	6.9 ± 6.7	
Male	7.5 ± 6.6	0.42
Female	6.8 ± 6.1	
> 13 week wait time	7.3 ± 6.4	0.94
< 13 week wait time	7.2 ± 6.5	

Note: Urban participants were defined as those living in the city of Winnipeg, Canada, while rural participants were those individuals living outside of the city limits

## Discussion

This study investigated whether there is an association between ECOHIS scores and surgery wait times for children undergoing dental treatment for S-ECC under GA in Winnipeg, Canada. We observed weak and non-significant associations between children’s wait times and ECOHIS scores. The lack of evidence for significant correlations between wait times and ECOHIS scores supports our original hypothesis that there is no present association between children’s wait times for pediatric dental rehabilitative surgery and their ECOHIS scores. This is understandable since ECOHIS is not routinely performed and scores are currently not used to prioritize children for pediatric dental surgery. However, with healthcare reforms underway in Manitoba, there is a recognition for a central intake for referrals and enhancing referral information to include validated measures of pain and quality of life that would assist pediatric dentists in prioritizing their cases for GA. The addition of ECOHIS, or elements of ECOHIS, into standardized referral forms is even more urgent now given the disruption that COVID-19 has had on dental care, including rehabilitative surgery performed under GA. Since the COVID-19 pandemic led to the temporary cancellation of elective pediatric dental surgery under GA, including the treatment of ECC, now is a fitting time to implement new processes to improve and ensure timely access to rehabilitative care and manage surgical waitlists [17].

To our knowledge, this study is the first to begin to examine ECOHIS scores in relation to wait times for dental treatment for young children. There is a dearth of research that examines the association between prolonged wait times and the ECOHIS specifically as a measure of the burden of dental disease. However, greater research into this relationship would hopefully show that a practical and validated tool such as ECOHIS would be an important metric to help triage and prioritize cases for surgery under GA.

Our results differ from the results of other studies that have examined different measures of the burden of dental disease in relation to prolonged waits for surgical intervention. A 2010 Toronto study examining the progression of tooth decay and stages of treatment needs over wait times for children undergoing elective dental surgeries under GA at The Hospital for Sick Children found that the increasing severity of dental disease was significantly associated with prolonged waits for surgical procedures (*p* < 0.001) [18]. Those authors proposed a prioritization system for their own tertiary care centre. Another study published in 2015 showed that problems such as pain, sleepless nights, and missed school were exacerbated by extended wait times for surgery [19]. North et al. also reported similar findings and found that children suffered from greater pain and disruption in their life when they were required to wait longer for dental extractions under GA [20]. These studies generally revealed a significant increase in the burden of dental disease with extended wait times for intervention or surgery [18–20]. This may be because children with greater caries burdens also experience more complex social needs and barriers, and medical histories that make it more difficult for them to access care in an expedited manner. It should be pointed out that the ECOHIS is context-dependent, and results may vary greatly depending on the location and culture in which it is being used.

It is important to note that the sample of children in the above-mentioned studies are different from the sample of children examined in this study. The investigators of this project specifically recruited children that were under 72 months of age, and that fell into the American Society of Anesthesiologists (ASA) physical classification system categories of ASA-1 (normal healthy patients) or ASA-2 (patients with mild systemic disease) [15, 21]. Children in all three of the studies discussed above were older, more medically compromised, and a more diverse group. Age may have affected the reporting of the burden of dental disease, as older children may have been able to describe OHRQL problems (to investigators and their parents) better than those under 72 months. These potential differences may have had a significant impact on questions in the CIS, especially.

This study was initiated with the hope of using findings to help justify and inform the adoption and implementation of ECOHIS as part of the prioritization process for children waiting for dental rehabilitative surgery in Manitoba. There are several requirements that would have to be met for ECOHIS to be effective in the prioritization process. First, ECOHIS should be able to accurately describe the impact of S-ECC on children and their families. This requirement has been met as ECOHIS is a validated tool that can identify whether or not dental disease is having a negative impact on OHRQL [16, 22–24]. Secondly, ECOHIS would ideally be able to detect significant differences in the severity of dental disease over time so that the prioritization of children waiting for surgery would be meaningful. This means that children waiting longer for surgery would theoretically be at risk for higher ECOHIS scores and a greater need for timely treatment than children with shorter waits for surgery. While trends of increasing ECOHIS scores were found for CIS and total scores in comparison with longer wait times, they were not significant. Finally, children with higher ECOHIS scores (perhaps in the CIS) and a greater need for surgical intervention would receive more timely care than children with lower ECOHIS scores (i.e., they would be granted shorter wait times at the time of the decision to treat S-ECC) [7, 14, 25].

ECOHIS is a tool that could ultimately assist pediatric dentists in prioritizing their patients and the field referrals they are receiving from other providers. Using ECOHIS to help triage and prioritize cases for surgery under GA would help those at risk who cannot wait as long for care, or who have already waited too long for care, receive the treatment they need.

The large proportion of children with dental benefits from the NIHB program suggests that many registered First Nations children are receiving dental surgery in the province, which is consistent with findings that the rates of dental surgery for ECC are particularly high in northern Manitoba (up to 116/1000 children) where many First Nations, Métis, and Inuit communities are located, and in neighbourhoods with a high proportion of Aboriginal Canadian persons (84.5/1000 children) [11, 12, 26]. The fact that almost all participants said that their child’s dental problems and dental treatments never had a financial impact on the family could be attributed to almost everyone in the study having some form of dental insurance that covered all or part of the family’s dental expenses.

Differences in ECOHIS FIS scores between families living in rural areas of Manitoba and families living in the city of Winnipeg are not surprising. Children residing in rural areas face several issues which influence access to care and their oral health, including a lack of dental professionals in their own community, and the need to travel outside their community for basic oral care [27, 28]. These barriers, coupled with the fact that children from rural regions have higher rates of dental surgery for ECC than urban dwelling children (31.2/1000 children vs. 9.8/1000 children in 2013), may cause greater distress for parents and caregivers, and may explain why FIS scores are significantly higher among those living in rural areas of Manitoba.

This study has several limitations. We used a convenience sample of 200 children with parents who were willing to participate in the study and we did not have controls who were not scheduled to undergo dental surgery under GA. ECOHIS data were only available for 174 children, meaning that we just failed to meet our desired sample size needed for adequate statistical power. As Grant et al. noted, in some instances a proxy (parent/caregiver) was required to answer the ECOHIS questions on behalf of the participant child and parent/caregivers’ responses may not have been reflective of the child’s own health rating [15, 29]. Furthermore, there is a concern that omitting the impact of caries severity at the time of recruitment may have influenced results. We did not have access to all of the decayed, missing and filled teeth (dmft) scores of participating children, which limited our analysis. As previous studies involving the ECOHIS have shown, children with more or less carious lesions in different stages of progression can have different OHRQL reports and different ECOHIS scores [30, 31]. The lack of evidence for higher ECOHIS scores in relation to longer wait times could be explained by the fact that, by the time of the decision to treat and at the time of recruitment, patients’ S-ECC-related burden or OHRQL may have approached maximal severity for their respective conditions [17]. Another limitation is that ECOHIS is not yet incorporated or adopted into the referral process or prioritization process for slating children for dental surgery. Missing wait times data for some children is also a limitation. Wait times may be subject to a few conditions, especially whether dentists’ offices are entering information into central waiting lists in a timely fashion. This provides rationale for a central intake process to manage waitlists for dental surgery under GA.

This study also has several strengths. The fact that we had a good sample of children recruited on the day of their surgery is a strength of this study. Another strength is that ECOHIS is a validated tool for measuring OHRQL. The high proportion of registered First Nations and rural participants recruited intro the study is notable. Representation from these groups is important as they experience some of the highest rates of pediatric dental surgery in Canada and face greater barriers in terms of accessing oral care to prevent and treat S-ECC [11, 12, 26–28]. Research pertaining to potential improvements to access to dental care could benefit these groups the most in the future. This was the first time that the ECOHIS was examined in relation to dental surgery wait times in Canada. It was also the first time that the ECOHIS was examined as a potential prioritization tool for children waiting for dental rehabilitation under GA. This study is novel and will hopefully set a precedent for future research on prioritization strategies to help children with S-ECC waiting for treatment under GA. Our next phase in this program of research is to study the potential for ECOHIS to be used in prioritization processes for children waiting for dental rehabilitative surgery. Adopting a measure of OHRQL in addition to caries scores may be helpful in giving children with the greatest negative impacts more timely access to rehabilitative care.

## Conclusion

As expected in this sample of children where measures of quality of life are not presently used to prioritize them for dental surgery, no significant associations were observed between ECOHIS scores and pediatric dental rehabilitative surgery wait times. Since the ECOHIS is a validated tool to measure OHRQL it could, however, still be used to assist in prioritization strategies, especially when there are considerable wait lists for surgery. As discussed above, ECOHIS scores tell us about a child’s OHRQL. Children with higher ECOHIS scores likely have more negative impacts and theoretically could be prioritized for surgery over children with lower ECOHIS scores. Further research is needed to investigate whether the implementation of ECOHIS to help prioritize children for surgery leads to improved wait times for those most in need of timely access to dental care under GA.

## Data Availability

The datasets used and analysed during the current study are available from the corresponding author on reasonable request.

## Abbreviations

ECC: Early Childhood Caries
S-ECC: Severe Early Childhood Caries
ECOHIS: Early Childhood Oral Health Impact Scale
OHRQL: Oral Health-Related Quality of Life
GA: General Anesthesia
PART: Patient Access Registry Tool
CI: Confidence Interval
CIS: Child Impact Section
FIS: Family Impact Section
WRHA: Winnipeg Regional Health Authority
P-CATS: Pediatric Canadian Access Targets for Surgery
ASA: American Society of Anesthesiologists
